# Barriers and facilitators for caregiver involvement in the home care of people with pressure injuries: A qualitative study

**DOI:** 10.1371/journal.pone.0226359

**Published:** 2019-12-23

**Authors:** Francisco José García-Sánchez, Vicente Martínez-Vizcaíno, Beatriz Rodríguez-Martín

**Affiliations:** 1 University of Castilla-La Mancha, Department of Nursing, Physiotherapy and Occupational Therapy, Faculty of Nursing, Ciudad Real, Spain; 2 University of Castilla-La Mancha, Health and Social Research Center, Department of Nursing, Physiotherapy and Occupational Therapy, Faculty of Nursing, University of Castilla-La Mancha, Cuenca, Spain; 3 Universidad Autónoma de Chile, Faculty of Health Sciences, Santiago, Chile; 4 University of Castilla-La Mancha, Department of Nursing, Physiotherapy and Occupational Therapy, Faculty of Health Sciences, Talavera de la Reina, Toledo, Spain; University of Auckland, NEW ZEALAND

## Abstract

**Aim:**

To explore the barriers and facilitators perceived by home caregivers regarding their involvement in the home care of people with pressure injuries.

**Background:**

Although home caregivers are key in the process of caring for people with pressure injuries, little is known about their perceptions regarding their involvement in the same.

**Methods:**

A qualitative study based on grounded theory involving a theoretical sample of 15 home caregivers of people with pressure injuries within the health district of Puertollano, Spain.

**Results:**

This study identified three barriers (feminization of care, necessary life adaptations as a home caregiver, and the organization of health services) and three facilitators (the perceived family duty for caring, willingness to provide care, and satisfaction with the care received on behalf of primary care services) associated with caregiver involvement in the home care of pressure injuries.

**Conclusions:**

The care of a person with pressure injuries is perceived as a duty and requires important adaptations affecting the home caregiver’s personal, social and work life. The emotional closeness and trust that develops between a patient and the primary care staff equals an involvement which, in turn, also has positive results for both the home caregiver and the patient.

## Introduction

Pressure injuries (PI) are a major burden for patients, home caregivers and the health system overall [1]. These are defined as injuries affecting the skin and/or underlying tissues as a result of pressure, or pressure in combination with shear [2]. In Spain, the prevalence of PI is 7.9% in hospitals, 13.4% in long-term care facilities and 8.5% among patients cared for at home via primary care services (PCS) [3]. These data are similar to those reported in France (8.1%) and slightly lower than other Northern European countries, such as Norway (14.9%), Sweden (18%), Belgium (21.1%) or Great Britain (21.5%) [4–6]. In the Americas, the prevalence of PI in the hospital setting is approximately 22.9% in Canada [7] and 10% in Brazil [8]. Most frequently, PI appear in older people, with a higher prevalence in people over the age of 70 years [5].

Although PI are a significant health problem among people living in long-term care facilities, most PI are cared for at home [9,10]. In southern European countries such as Spain, Italy or Greece, families have been traditionally involved in the care of older people and those with chronic disabling pathologies, in an attempt to allow the family member to live at home for as long as possible [11]. It has similarly, been recognized that the presence of a home caregiver helps avoid or delay institutionalization [12]. In this setting, the home caregivers play an essential role in caring for older people, especially those with chronic pathologies [10]. As a result, the achievement of care objectives is strongly dependent on their involvement, since home caregivers constitute the nexus between the patient and the primary care professionals [11]. Therefore, the involvement of the home caregiver is essential for the successful prevention and treatment of PI, since the effective collaboration and involvement of the home caregiver in the treatment of PI directly increases the possibility of a recovery [10].

Some studies have highlighted the importance of understanding and analyzing the expectations of both patients with PI and their home caregivers, regarding the care the former receive, in order to improve health practices [13–15]. In this sense, a systematic review of studies conducted in a hospital setting reported that, in order to optimize the management of PI and other chronic injuries, improved communication among professionals, patients and their home caregivers was necessary [16]. The importance of including the patients’ preferences with regards treatments was also highlighted [14,17]. Likewise, the shared responsibility during clinical decision making, the exchange of information and knowledge, and involving home caregivers in care, have been proposed as being factors that improve the process of care for PI [18]. This is associated with patients’ perceptions of a greater quality of care and a lower risk of adverse effects [19]. In order to involve patients in shared decisions and to consider their interests and opinions, it is necessary to implement a participatory management model enabling managers to adopt a flexible leadership model whereby autonomy is shared by all stakeholders [20].

Although some studies have explored the experiences of home caregivers of patients with PI among institutionalized patients [14], to our knowledge, few studies have analyzed this phenomenon from the perspective of community dwelling patients [21,22]. This perspective could help improve the care of patients with PI [23] and adjust interventions according to the needs of each patient [24]. Thus, this study was aimed at exploring the barriers and facilitators perceived by home caregivers regarding their involvement in the home care of people with pressure injuries.

## Methods

A qualitative study based on grounded theory methodology, involving a theoretical sample of family members who were, or had been, the home caregivers of people with PI [25,26]. Semi-structured interviews were used for data collection. This inductive method was used as it enabled the possibility of obtaining an in-depth understanding of the phenomenon, leading to a theoretical explanation of the barriers and facilitators of the home care of PI, based on an analysis of the participants’ experiences.

### Sampling and data collection

The study sample was based on patients attending four primary care health centers in Puertollano, Spain. The following inclusion criteria were used for the selection of participants: 1) home caregivers over 18 years of age and who did not receive any financial compensation for caring for a person with a PI; and 2) home caregivers of patients belonging to the healthcare district of Puertollano (Ciudad Real). This study excluded home caregivers who were unable to communicate in Spanish and those with cognitive decline or any other pathology that hampered participation in this study.

Based on evolving theoretical constructs of the theoretical sampling, additional events, activities or incidents were considered. Theoretical construct helping to explain how and why certain phenomena behave the way that they do. Thus, a continuous comparison evolved between these and the theoretical constructs [27]. In order to gather a wide variability of discourses from participants, theoretical sampling was employed to include home caregivers of both sexes, different ages and sociodemographic characteristics (marital status, relationship with the person with pressure injuries, work status, level of education) until reaching the criteria of theoretical saturation of data, at which point, continuing to increase the sample would not have provided any new analytical concepts [26] (Table 1).

**Table 1:** Characteristics of the home caregivers of people with pressure injuries who participated in the interviews.

Variables		Men	Women
Age of the caregiver	25–45 years	0	3
	46–65 years	1	3
	66–85 years	4	3
	>85 years	0	1
Marital status	Single	1	3
	Married	1	4
	Widow/er	3	3
Relationship with the person with PI	Partner	3	3
	Son/daughter	2	4
	Grandchild	0	1
	Sibling	0	1
	Neighbor	0	1
Level of education	Primary education	5	7
	Secondary education	0	1
	University studies	0	2
Work status	Unemployed	1	1
	Housework	0	2
	Employed outside the home	0	3
	Retired	4	4

Semi-structured interviews were used for data collection. The interviewer (main researcher) carried out a reflexive process in order not to influence the interviews. Reflexive process helps to improve the quality of the research. Thus this process tries to avoid biases, theoretical predispositions, preferences and so forth, by registering the self-reflection relfexive processes [28]. The interviews were semi-structured since open questions were used to promote the participants’ discourse, giving them the opportunity to describe their perspectives and the subsequent interpretation of their experiences. Moreover, we carried out a preliminary pilot study to verify that the open questions formulated by the interviewer did not influence the participants’ speech. The interviewer used an interview theme guide, based on themes that could appear during the interviews (S1 Appendix).

The interviews lasted between 45 and 60 minutes and took place at the homes of the participants between the years 2015 and 2016. All interviews were audio recorded using a digital recorder. After being anonymized, verbatim transcriptions of the interviews were performed, guaranteeing confidentiality in the treatment of data at all times.

### Ethical considerations

This study complies with the Helsinki Declaration and the legislation for the protection of personal data. The Ethical Committee of Clinical Research of Ciudad Real Hospital (Spain) approved this study (record number n° 11/2014) and authorization was obtained by the managers of the participating health clinics. All participants signed the informed consent after a full explanation of the research project.

### Data analysis

Following the principles of grounded theory, specific concepts were identified during the analysis process. Additionally, the participants voiced their feelings regarding the home care of PI. The verbatim transcriptions of the interviews were analyzed to explore the experiences of the home caregivers regarding the home care of PI, thus obtaining a theoretical explanation of the phenomenon under study. Two researchers who were experts in qualitative methods independently analyzed the interview transcripts, after which they discussed their results and reached a consensus [29]. Any discrepancies were resolved by a third researcher [30].

The constant comparative method was used during the process of data analysis and coding (open, axial and selective). This method allowed to us to generate a theory to explain the phenomenon under study based on the participants’ perspective. For this purpose, a continuous comparison of the data was reviewed in order to elaborate and compare novel study categories [26]. The researchers shared the emerging codes with operational memos and agreed on the categories and subcategories. They also created and shared a new hermeneutical unit for the project using Atlas-ti software. S2 Appendix shows examples of operational memos, codes, categories and subcategories.

During the analysis of the interview transcripts, and the review of the literature and emerging theoretical ideas and codes, each finding was compared with existing findings arising from the analysis [29]. Open, axial and selective coding and theorization was part of the data analysis process [26,31]. Open coding methods enabled the researchers to break down the data into smaller meaningful units, and then organize these into categories. Subsequently, the process of axial coding related the resultant categories and subcategories and, finally, selective coding led to the development of a theory to explain the phenomenon under study from the point of view of the participants [32,33].

The ATLAS-Ti 7.5.13 computer program was used as data analysis software during data analysis and coding.

### Validity

To guarantee the reliability and validity of the study conclusions, the Morse qualitative research principles, were followed. These ensured methodological coherence, sampling sufficiency, the development of a dynamic relationship between sampling, data collection and analysis, theoretical thinking, and theory development [34]. Methodological coherence was guaranteed through consistency between the research objectives and the components of the method. The sample was comprised of a sufficient number of participants, thus guaranteeing efficient data saturation. Data were collected and analyzed simultaneously in such a way that the iterative interaction between data and analysis was essential to achieve reliability and validity. Thinking theoretically meant constantly checking the entire process. Furthermore, the participants were given the opportunity to validate the content of the interviews based on the verbatim transcripts obtained. Finally, data triangulation was performed when conducting interviews with people with different characteristics according to the variables of interest for the study. Researcher triangulation was ensured by conducting data analyses independently by two researchers with experience in qualitative research and with different backgrounds (nursing, anthropology), and who subsequently agreed on the results. In case of disagreement, a third expert researcher in the field of public health intervened.

## Results

Theoretical sampling continued until 15 home caregivers of people with PI were recruited to the study. Three main barriers emerged from the data regarding the involvement of home caregivers in home care: 1) feminization of care; 2) necessary life adaptations as a home caregiver; and 3), barriers related to the organization of the care provided on behalf of the health services. Conversely, the three elements that facilitated this involvement were: 1) the influence of the perceived duty for a home caregiver to provide care; 2) the willingness of the individual to become involved in the care of PI and 3) satisfaction with the care received on behalf of the Primary Care Services and the nurses.

The results, themes, categories and codes which explain the perceptions of the home caregivers are summarized in Tables 2 and 3. Furthermore, a selection of the most representative quotes is provided in the description of results.

**Table 2:** Perceived barriers for the involvement of home caregivers in home care.

Feminization of care	Limited male involvement in care
Necessary life adaptations as a home caregiver	Personal adaptations
	Family adaptations
	Work adaptations
Barriers related to the organization of care on behalf of health services	Hospital work systems
	Lack of hospital staff
	Staff shifts

**Table 3:** Perceived facilitators for the involvement of home caregivers in care.

The perceived duty for a home caregiver to provide care	Influence of sociocultural aspects	
	Satisfaction with the duty fulflled	
	Commitment and loyalty towards the partner	
Willingness to be involved in pressure ulcer care	Wound care and treatments	Detection of complications
		Continuity of care
	Satisfaction with participation in the care of the pressure ulcer	
Satisfaction with the care received on behalf of the primary care services and nurses	Trust	
	Developing close relationships	

### Perceived barriers for the involvement of home caregivers in home care

#### 1. Feminization of care

The traditional association between gender and the role of caring emerged from the participants’ narratives, especially regarding the association of care with the female gender. Thus, although certain narratives described a minimal involvement of men in the role of the home caregiver, even in these minor cases, the participants highlighted the difficulties encountered by the male caregivers due to the perceived traditional gender roles:

*“I don’t know, perhaps women are more able to care, compared to men”*.

(Main Caregiver (MC).10).

Participants highlighted the little or null male participation in the care of people with PI at home, justifying this with aspects related to the culture and educational differences according to each gender.

“I have never done anything at home, I have always worked in the field, and she took care of the children and the house.”

(MC.13).

#### 2. Necessary life adaptations as a caregiver for a person with a pressure injury

Independent of gender, the home caregivers highlighted that the dedication required to care for a person with a PI entails a considerable effort, and it was necessary for them to adapt their personal, family and work life.

**Personal adaptations**: These include changes in the place of residence, as well as changes affecting lifestyle habits, and personal leisure time in order to care for the other person:

*“When I realized that the decline was already very significant, I came here permanently and, of course, I changed my life and my workplace, I left my son in Catalonia”*.

(MC 10).

**Family adaptations**: This included changes in family dynamics in order to care for the other person, for example, lifestyle modifications:

*“Of course, I had to change things, first I had to go back to my parents’ house to stay here, especially at the beginning. In a few months, I left my partner on his own”*.

(MC.2).

**Work adaptations**: The participants had to request changes in work shifts, work leave, reductions in work hours or greater flexibility in their workday:

*“At my work, I had to change shifts etc., in order to spend more time [performing care duties]. First, I used up my holidays. I moved them forward and then I requested to work part time”*.

(MC.2).

#### 3. Barriers related to the organization of the care provided by the health services

Among the perceived obstacles for participants’ involvement in care, home caregivers highlighted differences in the organization of care according to the care services used. For example, some hospitals disallowed caregivers to be present during the performance of care procedures. This hampered their participation during care, and was something that did not occur at the patient’s home, where the home caregiver was always present:

*“At home, I am always willing to lend a hand, but at hospital they don’t let you stay when the doctor comes by or when they care for the lesion, so you can’t do anything”*.

(MC. 15).

### Perceived facilitators for the involvement of home caregivers in care

#### 1. The perceived duty for caring for a family member

Most participants considered that the family should be the home caregiver of people with PI, perceiving this as a duty that is transmitted and inherited from parents to children.

**Influence of sociocultural aspects**: Taking care of a family member was prioritized by the home caregivers in comparison with other work or leisure related activities. In addition, the home setting was considered by the participants as being the optimal environment for caring:

“It seems like it is frowned upon if you don’t take care of your parents”

(MC.10).

*“I take care of my grandmother, I think that it’s better at home than anywhere else, however good that place may be”*.

(MC.8).

**Satisfaction with the duty fulfilled**: One of the reasons for caring was the personal satisfaction experienced by the home caregivers. Fulfilling the duty at hand was accompanied by feelings of peace and tranquility, with participants stating that they would do it again if they ever found themselves in the same situation:

*“Feeling calm and at peace. I think I did what I was supposed to with my mother and that is what matters. I would do it again and again. It was my obligation and I have been able to do it”*.

(MC.10).

**Commitment and loyalty towards the partner**: The perceived duty for caring was related to feelings of commitment and loyalty towards the partner:

*“I am very tired, I am very old, but I have to be with him, we have been together for over 60 years, and I am not going to leave him now”*.

(MC. 7).

#### 2. Willingness to become involved in the care of the pressure injury

**Wound care and treatments**: The home caregivers were involved in the care of PI by participating directly in the care procedures and other treatments (hygiene measures, postural changes, etc.,) performed by the health professionals who went to the home and following their instructions. This care was key to ensuring an appropriate continuity of care:

*“I helped the nurse by holding my mother, and I also learnt how to move her and wash her and sometimes I cared for her myself in a pretty rudimentary manner, but I did it and she liked it”*.

(MC. 2).

Thus, the existence of a fluid and appropriate communication between the family and the healthcare team was considered essential to facilitate the involvement of families in the provision of care:

*“I did what I could because I didn’t know anything about these things, I helped to move him, and I saw how he was doing, and I told them how his days and nights went and whether I noticed anything odd. I always communicated with them well and that’s good”*.

(MC. 5).

**Satisfaction with participation in the care of the pressure injury**: Participation in the care provided by professionals, especially regarding the care for patients with PI was perceived by participants as a reason for personal satisfaction:

*“The truth is you feel better if you see how it evolves and if it gets better you feel that your work is producing results and you are happy”*.

(MC. 10).

#### 3. Satisfaction with the care received on behalf of primary care services and the nursing professionals

Although the home caregivers expressed their satisfaction with the care provided by the nursing professionals, independent of the health setting where it was provided (primary care services or hospital context), they stressed the value of the close relationship, trust and availability associated with the nurses who came to their home:

*“Very good, all of them [the nurses], both at home and at hospital. They are very good professionals and they took care of her very well”*.

(MC. 13).

*“At home, the relationship is different and closer, the nurse is the same and spends whatever time is necessary, they provide you with explanations and listen to you”*.

(MC. 6).

## Discussion

Several hypotheses emerged from the results of this study which help to explain the perceptions of the home caregivers of people with PI, regarding the barriers and facilitators for their involvement in home care:

1. The willingness and interest of the home caregivers in collaborating with the primary care professionals during the treatments was considered to facilitate their subsequent involvement in the home care of the PI.
2. The family obligation towards care can be perceived as a facilitator linked to the traditional cultural aspects of caring among home caregivers in countries where there are less women who work outside the home.
3. The positive opinion regarding primary care services in terms of the development of close relationships, availability and trust, facilitated the participation of the home caregivers in the care of people with PI.
4. The lack of male involvement in care tasks was considered a barrier to becoming involved in the home care of PI.
5. The need to make major adaptations to one’s personal, family and professional life, in order to care for a family member, emerged as a barrier for home caregivers.
6. The poor organization of some care resources was considered a barrier that could hinder the participation of home caregivers.

Although several studies and international agencies recognize that the involvement of home caregivers in care is key for providing a quality care service [35,36], this is one of the few studies focused on analyzing the perceptions of the home caregivers on the elements that facilitate or hamper their involvement in the home care of people with PI. Due to the particular characteristics of the care model used in primary health services in Spain, we are unable to compare this with care provided in other countries [3].

In line with previous studies, the perceptions of the home caregivers of people with PI follow the prevailing model of countries with a more limited presence of women in the workforce, considering that home care for the elderly is a family obligation [37]. In addition, admitting the person with PI into a long-term care facility is perceived as a failure in the role of the home caregiver and as the last available option [38], considering the home as the best setting for care.

Another finding which reinforces previous reports, is that the care of a family member is almost exclusively a woman’s responsibility; a result that confirms the gender inequality that exists in the home caregiver’s role [39]. In this sense, the results of this study confirm the social tendency observed in southern Europe countries, namely that women generally assume a greater responsibility in the care of children, partners, and dependent family members [37]. Our analysis supports the fact that, in the family context, caring is almost exclusively considered to be a female role [39,40].

Additionally, the results of this research follow the line of previous studies revealing that, in order to provide care, the home caregivers must make changes to their personal, work and social lives including, at times, situations which may end up negatively affecting their quality of life. Nevertheless, home caregivers continue carrying out these tasks, which are perceived as being their duty, while highlighting their own personal satisfaction in doing so, performing a job that is well done, and perceiving the benefits that this provides to their family member [40–42].

The participants in this study spoke of feelings of collaboration and proximity among themselves and the home based primary care services (mostly provided by nurses), confirming the positive opinions voiced by home caregivers regarding the care provided on behalf of the primary care services. In contrast, concerning the hospital context, although the participants were generally satisfied with the care provided by the nursing professionals, they encountered difficulties for becoming involved in the care provided at hospitals.

This research provides a novel approach for the home treatment of PI on behalf of primary care services by including the perceptions of the home caregivers of people with PI. Besides, this research sheds light on the importance of knowing the patients’ preferences and their previous knowledge regarding their health in order to actively participate in shared decision-making [43]. The findings of this study provide nurse managers with valuable information which may be incorporated into quality of care improvement programs.

### Limitations

Qualitative methods do not enable the generalization of findings, however this study provides an in-depth understanding of the perceptions of the home caregivers of people with PI, opening new paths for improving care, which was the aim of this research [44]. Although the size of the sample can be considered a limitation, the use of a theoretical sample ensured the inclusion of an important representation of participants with a variety of characteristics. Moreover, data saturation was reached [33].

Due to the inexistence of similar studies in this field, this research has not been influenced by former studies. Grounded theory, and the use of the constant comparison method enabled the maintenance of the theoretical sensitivity during each phase of the study. During this process, the method of constant comparisons was used, which involved the comparison and continuous review of the data, allowing the research team to develop and compare new study categories and seek a theory to explain the phenomenon of the study from the point of view of the participants. Therefore, there was a constant back and forth between the interview transcripts, the theoretical ideas regarding the codes, their transcripts and the literature review. All this enabled the maintenance of the theoretical sensitivity during each phase of the study while, at the same time, enabling the generation of theories based on the data, avoiding preconceived ideas from other studies or from pre-existing theories.

## Conclusions

The involvement of home caregivers in the care of PI improves their satisfaction with the care provided. The closeness, trust and availability of primary care staff are factors that facilitate the involvement of home caregivers in care. Female family members are the most frequent home caregivers of patients with PI in the home. The care of a person with PI requires important adaptations in the personal, social and work life of home caregivers. Despite this, the feeling of satisfaction for having fulfilled their duty with their family member is highlighted. Institutionalization in a long-term care facility is experienced as a failure in the role of the home caregiver and is considered as the last therapeutic option for people with PI, as home caregivers believe that the home is the best setting for care. A better understanding of both the barriers and the facilitators of care provides valuable information towards promoting a more comprehensive approach to the treatment of PI, and providing nurse managers with valuable information that can be incorporated into programs to improve the quality of PI care.

## Supporting information

S1 Appendix Interview theme guide. (DOCX)

S2 Appendix Memo and coding process examples. (DOCX)
