# Supplementary material for: Barriers and facilitators for caregiver involvement in the home care of people with pressure injuries: A qualitative study
Source: PLoS One. 2019 Dec 23;14(12):e0226359. doi: 10.1371/journal.pone.0226359 (PMC6927621; doi:10.1371/journal.pone.0226359)
Supplement: S1 Appendix — (DOCX) [file pone.0226359.s001.docx]

**S1 Appendix.- Interview theme guide used during interviews with the home caregivers of people with pressure injuries.**

Theme 1:

Personally experiencing the care of a person with pressure injuries

Theme 2:

Main concerns and doubts regarding caring for a person with pressure injuries.

Theme 3:

Main changes in everyday life, at your work and changes in the family dynamics after the appearance of the injury

Theme 4:

Thoughts on the care received by the nurses who cared for the pressure injury

Theme 5:

Areas for improvement regarding the treatment of pressure injuries
