# Supplementary material for: Barriers and facilitators for caregiver involvement in the home care of people with pressure injuries: A qualitative study
Source: PLoS One. 2019 Dec 23;14(12):e0226359. doi: 10.1371/journal.pone.0226359 (PMC6927621; doi:10.1371/journal.pone.0226359)
Supplement: S2 Appendix — (DOCX) [file pone.0226359.s002.docx]

**S2 Appendix**

**2. 1 Memo example: Willingness to be involved in pressure injury care.**

When wound care and treatments such as the detection of complications or continuity of care and satisfaction with participation in the care of the pressure injury appeared in the participants’ speech.

**2.2 Example of coding process**

**Perceived facilitators for the involvement of home caregiver in care.**

| **Codes** | **Categories** | **Subcategories** |
| --- | --- | --- |
| The perceived duty for a family member to provide care | Influence of sociocultural aspects |  |
|  | Satisfaction with the duty fulfilled |  |
|  | Commitment and loyalty towards the partner |  |
| Willingness to be involved in pressure injury care | Wound care and treatments | Detection of complications |
|  |  | Continuity of care |
|  | Satisfaction with participation in the care of the pressure injury |  |
| Satisfaction with the care received on behalf of the primary care services and nurses | Trust |  |
|  | Developing close relationships |  |
